# Detection of VEGF-A_xxx_b Isoforms in Human Tissues

**DOI:** 10.1371/journal.pone.0068399

**Published:** 2013-07-31

**Authors:** David O. Bates, Athina Mavrou, Yan Qiu, James G. Carter, Maryam Hamdollah-Zadeh, Shaney Barratt, Melissa V. Gammons, Ann B. Millar, Andrew H. J. Salmon, Sebastian Oltean, Steven J. Harper

**Affiliations:** 1 Microvascular Research Laboratories, School of Physiology and Pharmacology, University of Bristol, Bristol, United Kingdom; 2 School of Clinical Sciences, Bristol, United Kingdom; INSERM, France

## Abstract

Vascular Endothelial Growth Factor-A (VEGF-A) can be generated as multiple isoforms by alternative splicing. Two families of isoforms have been described in humans, pro-angiogenic isoforms typified by VEGF-A_165_a, and anti-angiogenic isoforms typified by VEGF-A_165_b. The practical determination of expression levels of alternative isoforms of the same gene may be complicated by experimental protocols that favour one isoform over another, and the use of specific positive and negative controls is essential for the interpretation of findings on expression of the isoforms. Here we address some of the difficulties in experimental design when investigating alternative splicing of VEGF isoforms, and discuss the use of appropriate control paradigms. We demonstrate why use of specific control experiments can prevent assumptions that VEGF-A_165_b is not present, when in fact it is. We reiterate, and confirm previously published experimental design protocols that demonstrate the importance of using positive controls. These include using known target sequences to show that the experimental conditions are suitable for PCR amplification of VEGF-A_165_b mRNA for both q-PCR and RT-PCR and to ensure that mispriming does not occur. We also provide evidence that demonstrates that detection of VEGF-A_165_b protein in mice needs to be tightly controlled to prevent detection of mouse IgG by a secondary antibody. We also show that human VEGF_165_b protein can be immunoprecipitated from cultured human cells and that immunoprecipitating VEGF-A results in protein that is detected by VEGF-A_165_b antibody. These findings support the conclusion that more information on the biology of VEGF-A_165_b isoforms is required, and confirm the importance of the experimental design in such investigations, including the use of specific positive and negative controls.

## Introduction

Vascular Endothelial Growth Factor-A is generated as multiple splice isoforms using alternative splice sites within exons 6, 7 and 8 in normal and pathological tissues [1], [2]. Alternative splicing of the terminal exon, exon 8 gives rise to two families of isoforms, VEGF-A_xxx_a and VEGF-A_xxx_b, which have the same number of amino acids but different C terminal sequences [3]. The differences between these two families of isoforms lie in the deletion of 66 nucleotides from the beginning of exon 8 arising from a 3′ alternative splice site. The VEGF-A_xxx_b family was serendipitously discovered in 2002, after the amplification of PCR products using primers in the 3′ untranslated region of exon 8 of cDNA generated from multiple normal human kidney samples collected at the time of nephrectomy and frozen. It was notable that this product was less commonly found in the renal carcinomata from the same whole organ samples [3]. Since 2002, in addition to the VEGF_xxx_b isoform first identified, VEGF-A_165_b, studies have also demonstrated the isoforms VEGF-A_121_b [4], VEGF-A_189_b [5] and VEGF-A_145_b [6]. Most of these studies have investigated expression in fresh human tissue, and most studies have found the VEGF-A_xxx_b mRNA to be downregulated in pathological conditions such as cancer [7], diabetic retinopathy [6], Denys Drash Syndrome (a condition caused by a mutation of the tumour suppressor gene WT1) [8], and retinal vein occlusion [9]. In contrast VEGF-A_165_b has been shown to be upregulated in systemic sclerosis [10] and in asthma [11]. The regulation of VEGF splicing has been investigated and it has been demonstrated that in epithelial cells, growth factor stimulation (e.g. by IGF) induces phosphorylation of the Serine Arginine Rich Factor 1 (SRSF1) by the kinase SR protein Kinase 1, enabling nuclear localisation of SRSF1 and binding to the VEGF pre-mRNA, facilitating splicing to the proximal splice site, and VEGF_165_a expression [12]. SRPK1 over-expression, for instance by removal of transcriptional repression in WT1 mutant cells [13], results in increased VEGF_165_a, whereas SRPK1 knockdown or SRPK1 inhibition increases VEGF_165_b expression. This is seen in patients with WT1 mutations, such as Denys Drash Syndrome [8]. In contrast activation of SRSF6 by phosphorylation from Clk1/4 downstream of TGF-β results in preferential VEGF_165_b expression [14]. This is seen in conditions where VEGF expression is high but angiogenesis is deficient such as systemic sclerosis [10].

VEGF-A_165_b expression has been demonstrated by RT-PCR (qualitatively [3] and quantitatively [15]), or by using antibodies raised to the unique 9 amino acid C’ terminal tail of VEGF-A_165_b/VEGF-A_189_b (CTRSLTRKD) by western blot [7], immunofluorescence [16] or isoform-family specific ELISA [7].

Alternative mRNA splicing is a process whereby more than one mRNA can be generated from a single gene. In many cases cells will produce multiple mRNA products from a single gene, and the relative ratios of those isoforms to each other will be dependent on the cell physiology and control of cell function both genetically and in response to environmental cues. For instance, cells may express both isoform families of an alternatively spliced gene and the relative levels of those mRNA species can vary according to environmental or genetic influences [17]. Assessment of VEGF-A_165_b mRNA expression can be carried out by RT-PCR amplification of cDNA template either using primers that detect both VEGF-A_xxx_b and VEGF-A_xxx_a isoforms, (i.e. isoform-common primers), or by isoform specific primers (see Methods).

Equally the assessment of the protein products of alternative spliced mRNAs requires the use of antibodies where the specificity of the antibody for the targets are controlled for. Alternative splicing is not as closely conserved across species, with estimates of 30–60% conservation of splicing events between human and mouse [18], [19], [20] compared with ∼80% conservation of gene orthologues [21]. One report estimates alternative 3′ splice events to be conserved between human and mouse in less than 20% of cases [18]. Moreover, different species can use different splicing paradigms to provide the same function. For VEGF-A_165_b it appears from published work that the proportion of VEGF-A that is the VEGF-A_xxx_b isoforms increases through the evolutionary tree from mice, through rabbits to monkeys and humans [22].

Both types of detection (protein and RNA) have inherent assumptions and technical issues that need to be addressed for the accurate assessment of these alternatively spliced isoforms. It is therefore critical that quantitation of alternative splicing products, including VEGF-A, uses an experimental design that allows for these assumptions. Therefore experiments that fail to detect VEGF-A_165_b expression can only support assumptions about VEGF-A_xxx_b isoforms if appropriate controls are used [23]. For instance, if experimental procedures are not shown to be capable of detecting VEGF-A_165_b mRNA or protein, then they cannot be taken as evidence of absence of VEGF_165_b without positive controls. Moreover, artefactual products may also be generated that could be mistaken for VEGF-A_xxx_b isoforms, but in the presence of positive and negative controls these artefacts can be identified. Finally, detection of proteins in mouse tissues using mouse monoclonal antibodies requires specific antibody controls. Using an anti-VEGF-A_165_b antibody in mice genetically lacking the appropriate sequences for VEGF-A_165_b splicing to occur [23] could provide evidence of antibody non-specificity, if appropriate negative controls are included. Absence of evidence rather than evidence of absence, results when controls are omitted. The detection of splice forms of the same gene does require attention to the detail of experimental design, as there are many parameters of the experimental procedures that may prevent detection of one isoform over another. To underline this we here provide some examples of why use of specific control experiments is of critical importance for investigation of VEGF-A_165_b expression.

## Materials and Methods

### Cells and Tissues

Human ARPE19 [24], PC3 [25], A375 [26], human lung fibroblasts [27], conditionally immortalised podocytes [13], colonic adenoma cell lines AA/C1 and the *in-vitro* transformed adenoma cell line 10C cells [28] were maintained as described previously. Primary human RPE cells were derived from donors from the Bristol eye bank, as previously described [14]. Use of animals was in accordance with UK legislation under a Home Office License approved by the UK government and University Bristol Ethics Committee. Commercially available cDNAs were obtained from either *Amsbio* (Kidney and Bladder) or *Primer Design* (Colon and Prostate).

### RT-PCR to Amplify Human VEGF-A_165_a and VEGF-A_165_b

Conventional PCR was used to detect VEGF-A_165_a and VEGF-A_165_b mRNA. cDNA was added to a reaction mixture containing: 2× PCR Master Mix (Promega, Southampton), isoform common primers (1 µM each) complementary to exon7a (5′-TTGTACAAGATCCGCAGACG–3′) and the 3′UTR of exon8b with a BamH1 site (underlined) for facilitation of cloning (5′-ATGGATCCGTATCAGTCTTTCCTG G-3′), made up to a total volume of 25 or 50 µl with DNase/RNase free water. This results in a PCR product for VEGF-A_165_b (129bp) that is 66bp shorter than for VEGF-A_165_a (195bp) All samples were run in parallel with negative controls (without reverse transcriptase; -RT) and positive controls (pcDNA3-VEGF-A_165_a and pcDNA3-VEGF-A_165_b). The reaction mixture was thermo cycled (PCR Express or PCR Engine; Biorad, Hemel Hemstead) 30 times, denaturing at 95**°**C for 60 seconds, annealing at 55**°**C for 60 seconds and extending at 72**°**C for 60 seconds. PCR products were then separated on 2.5% agarose gels containing 0.5 µg/ml ethidium bromide (BioRad) and visualized under an ultraviolet transilluminator (BioRad).

### RT-qPCR to Amplify Human VEGF-A_165_a and VEGF-A_165_b

RNA was isolated and quantified as previously described. Potential genomic DNA contamination was eliminated with DNase I enzyme digestion following the manufacture’s instruction with RQ1 DNase (Promega). Reverse transcription of DNase I – treated RNA with MMLV reverse transcriptase (Promega) was performed in the presence of both random primer (0.5 µg/1 µg of RNA) and Oligo dT (0.5 µg/1 µg of RNA) as previously described. Quantitative PCR (qPCR) was performed with a SmartCyclerII (Cepheid, Sunnyvale, CA, USA) q-PCR machine. The qPCR mix included 10 µl of SYBR Green Master (QIAgen,West Sussex, UK), 0.5 µM each of forward and reverse primers, plasmid DNA or cDNA from samples, and water to give a total volume of 20 µl. The isoform specific primers sequence were:

for VEGF-A_165_a amplification

forward primer 5′- GAGCAAGACAAGAAAATCCC-3′

reverse primer 5′- CCTCGGCTTGTCACATCTG-3′

for VEGF-A_165_b amplification

forward primer 5′- GAGCAAGACAAGAAAATCCC-3′

reverse primer 5′- GTGAGAGATCTGCAAGTACG-3′

qPCR cycle was 95°C for 15 mins, 45 cycles of 95°C for 30 secs, 60°C for 30 secs and 72°C for 30 secs, final extension at 72°C for 10 mins to end the PCR reactions. 10^−2^ to 10^−6^ ng of pcDNA3-VEGF-A_165_a and pcDNA3-VEGF-A_165_b plasmids were used as standards to calculate the amounts of VEGF-A_165_a/VEGF-A_165_b expressed in samples.

### Western Blotting (VEGF)

Protein extracted from primary RPE cells, ARPE-19, differentiated wildtype podocytes and differentiated DDS podocytes was denatured and 30 µg run on SDS-PAGE. Recombinant human VEGF-A_165_a and VEGF-A_165_b (R&D Systems) was run as positive controls (50ng of each). Proteins were separated using 4–20% precast gels (456–1094, BioRad) run for two hours at 90V and transferred to nitrocellulose membrane (15V, 10 minutes, 170–4150, BioRad). Membranes were blocked overnight in 2.5% (w/v) BSA in 0.1% (v/v) TBS-Tween at 4°C and co-immunoblotted with VEGF-A antibody (0.2 µg/ml, AF293NA, R&D) and VEGF-A_165_b specific antibody (1.2 µg/ml, MAB3045, R&D) in 2.5% BSA/TBST. Blots were washed with 0.1% TBS-Tween (5×5 mins) and co-immunoblotted with anti-mouse and anti-goat fluorescent antibodies (926–32212 and 926–68074 respectively @1∶10,000, Licor Biosciences) for 1 hour at room temperature. Following a final wash, blots were imaged using the Odyssey® Fc imaging system (Licor Biosciences).

### Western Blotting (Mouse IgG)

BalbC mice were killed by cervical dislocation under a UK Home Office License. Protein extraction from mouse tissues was carried out by liquid nitrogen homogenisation followed by RIPA buffer resuspension (5 µl/µg). Protein concentration was determined by Bradford Assay (BioRad) and 100 µg of protein denatured for PAGE with Laemmli Buffer and 5% β-mercaptoethanol for 5 minutes at 100°C. Recombinant human VEGF-A_165_a and VEGF-A_165_b (R&D systems), plus recombinant mouse IgG (DAKO) were run as controls (50ng of each). SDS PAGE was carried out as above. Membranes were then immunoblotted with either VEGF-A_165_b specific antibody (1 µg/ml, MAB3045, R&D) or no primary antibody in 2.5% BSA/TBST and left for 6 hours at 4°C. Blots were washed with 0.1% TBS-Tween (5×5 mins) and immunoblotted with anti-mouse fluorescent antibody (926–32212 at 1∶10,000, Licor Biosciences) for 1 hour at room temperature. Following a final wash, blots were imaged using the Odyssey® Fc imaging system (Licor Biosciences).

### Immunoprecipitation

Immunoprecipitation was carried out using the Millipore PureProteome system (Cat No. LSKMAGA02). Briefly whole cell lysate was prepared from 10C and AA/C1 cells from at least 2×10^6^ cells. Fifty µl of protein G or A beads suspension were washed with TBS-T (0.1%) and mixed with 500 µg cell extract and 5 µg of antibody (56/1 or AF293). The reaction was incubated with end over end rotation for 6 hours or O/N at 4°C. The mix was centrifuged for 30 seconds at 4°C and beads separated from the mixture using a magnetic stand. Beads were washed 3 times with TBS-T (0.1%) before adding the sample buffer. The beads were boiled in sample buffer for 5–10 minutes, cooled and the IP mix removed from the beads and used for SDS-PAGE and immunoblot or stored at −20°C until use.

## Results

The detection of the alternative splice isoforms in human tissues requires the use of appropriate positive and negative controls, and the interpretation of negative results needs to proceed with caution. Here we show some examples of the controls required for reasonable interpretation of results investigating VEGF isoform expression.

### Amplification of VEGF-A mRNA isoforms using isoform-common primers

**Figure 1:**
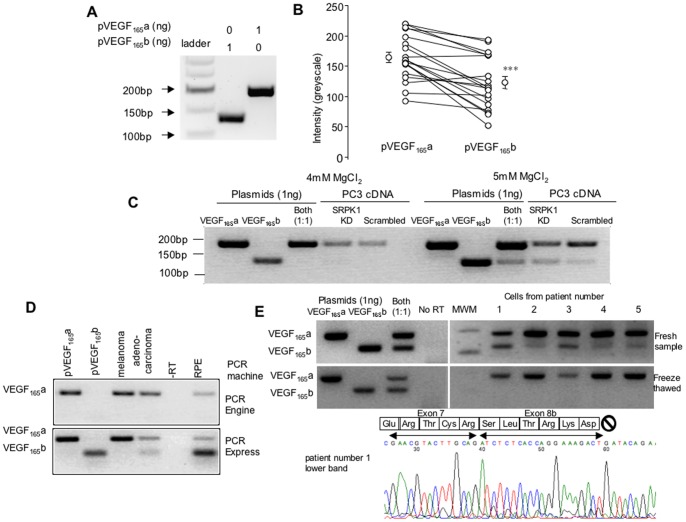
Positive controls are required to interpret lack of amplification of VEGF-A_165_b by competitive RT-PCR. A. Plasmids containing VEGF-A_165_b or VEGF-A_165_a sequence were amplified using primers in exon 8b and exon 7. Two different sized products were generated. B. Densitometric analysis of published RT-PCR gels using plasmids containing VEGF-A_165_b and VEGF-A_165_a and primers in exon 7 and exon 8b. 13/15 show higher intensity for VEGF-A_165_a. p<0.001 paired t test. C. Example of failure of amplification of the VEGF-A_165_b isoform. Two parallel PCR reactions were run on cDNA and plasmid DNA. On the first (at 4mM MgCl_2_) no VEGF-A_165_b was generated in the cDNA, or when both VEGF-A_165_a and VEGF-A_165_b plasmids were used as positive controls. In the second (at 5mM MgCl_2_) VEGF-A_165_b was generated from cDNA from PC3 cells where SRPK1 was knocked down, and when both templates were included. D. Example of failure of amplification of the VEGF-A_165_b isoform in one PCR machine (and failure of template), but not in a second machine. E. Example of failure of amplification of VEGF-A_165_b product after freeze-thawing of cDNA derived from fresh normal human lung fibroblasts. Chromatogram confirms VEGF-A_165_b sequence from lower band from top gel from patient sample in lane 1 (chromatograms from the other samples also confirmed VEGF-A_165_b sequence).

VEGF-A_165_b and VEGF-A_165_a isoforms differ according to the presence (VEGF-A_165_a) or absence (VEGF-A_165_b) of the proximal part of exon 8, termed exon 8a. Therefore isoform-common primers with a reverse primer situated downstream of the distal splice site (in the 3′UTR) and a forward primer in exon 7 will amplify both products. The PCR reaction is a non-linear amplification of cDNA, and the efficiency of the PCR reactions may be different for different PCR products, even when the primers are annealing to identical sequences, according to the GC content, secondary structure and initial post primer sequence of the PCR product. Figure 1A shows amplification of plasmids containing the VEGF-A_165_b and VEGF-A_165_a sequences using isoform common primers. Different size products are generated from the two plasmids, enabling VEGF-A_165_b (exon 8b product) to be distinguished from VEGF-A_165_a (exon 8a product) by size. There are a number of published studies using positive controls to ensure that amplification of the VEGF-A_165_b isoform is possible [8], [13], [15]. In many of these published PCR reactions the intensity of the VEGF-A_165_b band was lower than that of VEGF-A_165_a (figure 1B). We therefore strive to use positive and negative controls when investigating VEGF-A_165_b expression from cDNA generated from biological samples to ensure that the VEGF-A_165_b sequence can be detected under each specific PCR condition, and that the level of detection is similar to that for VEGF_165_a. This provides more confidence that a lack of product is reflective of reduced level of template, although unless the intensity of the PCR reaction products are similar, then a lack of product can only infer reduced template not its absence. One example of this is given in figure 1C. We altered the Mg^2+^ concentration of the PCR reaction while investigating expression of VEGF-A_165_b in prostate cancer cells. Figure 1C shows that, in the presence of 4mM MgCl_2_ VEGF-A_165_a but not VEGF-A_165_b expression was seen in PC3 cells treated with either control or SRPK1 specific shRNAi (recently shown to regulate VEGF splicing) [12]. However, under these conditions, the PCR did not amplify VEGF-A_165_b from a plasmid containing the VEGF-A_165_b sequence when in the presence of the VEGF-A_165_a plasmid. When the Mg^2+^ concentration was 5mM, the cDNA from PC3 prostate cancer cells showed weak VEGF-A_165_b expression in wild type PC3 prostate cancer cells, but VEGF-A_165_b was clearly upregulated by knockdown of SRPK1 compared with the scrambled control siRNA. Under these conditions the positive control cDNA was amplified. This does not suggest that 5mM Mg^2+^ is required (we regularly usually use 2.5mM MgCl_2_ to amplify VEGF cDNA), however this specific case illustrates that a single PCR reaction may preferentially amplify VEGF-A_165_a, resulting in apparent lack of VEGF-A_165_b.

The value of using positive control cDNA to determine whether failure to amplify the VEGF-A_165_b product from cDNA is due to experimental artefact or absence of template is also illustrated in figure 1D. In this case, the same template and PCR mixture was used and PCR carried out in two different PCR machines (Hybaid PCR express and BioRad DNA Engine) with the same cycling settings. In one machine, there was no amplification of VEGF-A_165_b, but efficient amplification of the VEGF-A_165_a in the cDNA prepared from three different cell types. However, on a different PCR machine, using the same primers, same buffers and the same cycle settings, VEGF-A_165_b was clearly seen in RPE cells, but not in melanoma cells. A very faint band was seen in an adenocarcinoma cell line previously shown to express VEGF-A_165_b protein (by sandwich ELISA using a VEGF antibody raised against the N-terminal end of the protein to capture protein, and a C-terminal VEGF-A_165_b specific antibody to detect captured protein). Here we used positive controls (VEGF-A_165_a and VEGF-A_165_b sequences in expression plasmids) to make sure we could amplify the VEGF-A_165_b product. Without the appropriate controls, the conclusion drawn from the upper gel would be that VEGF-A_165_b is not expressed in RPE cells, whereas the lower gel clearly shows that VEGF-A_165_b is expressed.

Even in the presence of positive controls, the differential amplification of the pro-angiogenic isoforms needs to be taken into account when interpreting negative PCR results. Figure 1E shows a PCR from cDNA reverse transcribed from RNA extracted from normal human lung fibroblasts. Amplification of the positive controls was successful (although again note the poorer amplification of the VEGF-A_165_b template). In all five samples both VEGF_165_a and VEGF_165_b were amplified, although at different levels. The PCR products were sequenced and VEGF-A_165_b sequence was confirmed (an example chromatogram from the first sample is shown underneath). This sequence demonstrates that the bands are unquestionably amplifications of VEGF-A_165_b. A repeat PCR of this cDNA was carried out one month later after three freeze thaw cycles (as cDNA was used for other experimental procedures) and no VEGF_165_b band was seen (lower gel). Note that all samples came up less strongly than previously, but because the VEGF_165_a was preferentially amplified, no VEGF_165_b was seen in this second PCR. A subsequent PCR with more starting template again showed VEGF_165_b in these samples.

### Isoform-specific Amplification of VEGF-A mRNA and qPCR

**Figure 2:**
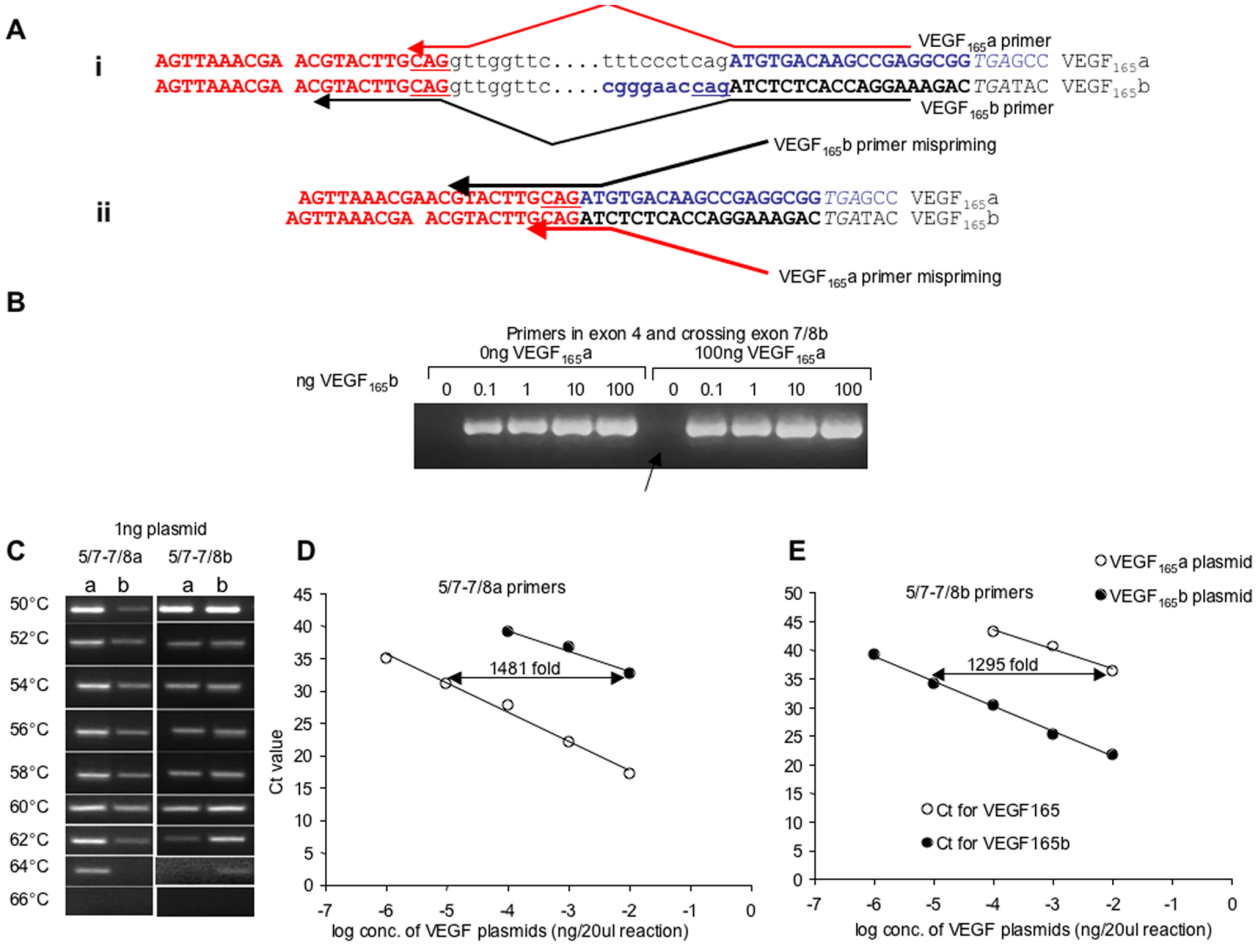
Isoform specific PCR requires positive controls to ensure specificity. A. Sequence of the VEGF 3′ exon sequence. (i) Exon 7 (red) contains the same last three nucleotides (underlined) as the last three nucleotides of exon 8a (blue, underlined), requiring specific PCR primers that extend into exon 7 (arrow). (ii) mispriming (VEGF-A_165_a -specific primers priming on VEGF-A_165_b, and VEGF-A_165_b -specific primers priming on VEGF-A_165_a) can occur both ways round if the conditions are not tested. B. Published control PCR gels demonstrating specificity of primer conditions. The original description of VEGF-A_165_b describing conditions at which VEGF-A_165_b is not misprimed in the presence of 100ng VEGF-A_165_a (lane highlighted by arrow), but still able to amplify 0.1ng VEGF-A_165_b. C. Annealing temperature dependence of the specificity of the isoform specific primers. Only at >62°C is specificity resolved. D. qPCR using VEGF-A_165_a specific primers on VEGF-A_165_a and VEGF-A_165_b plasmid E. qPCR using VEGF-A_165_b specific primers on VEGF-A_165_a and VEGF-A_165_b plasmid.

Use of isoform specific primers that cross the exon boundary between exon 7 and exon 8a or 8b requires conditions that result in specific priming. The nucleotide sequences at the junction between exon 7/8a and exon 7/8b share five nucleotides of identity around the splice site, two nucleotides in exon 8 and the three nucleotides immediately before the PSS and DSS (figure 2A). Specificity can be achieved by using primers that have specific nucleotide sequences extending past the three in exon 7 (if using reverse isoform specific primers) (e.g. ten nucleotides as shown in figure 2Ai) or past the three in exon 8 if using forward isoform specific primers [5]. Even in these cases, if the PCR conditions are not stringent enough, cross priming may occur (e.g. figure 2Aii). To avoid this specifically, previous publications using isoform specific primers, including the original description of VEGF-A_165_b [3], [6], [7], have included plasmid DNA from the other isoform as negative controls (figure 2B). Figure 2C shows that cross priming with primers illustrated in figure 2A occurs at lower annealing temperatures. At annealing temperatures greater than 62°C no cross-primed products were apparent under these conditions for RT-PCR. It is therefore clear that without the use of negative control DNA as well as positive control DNA it is not possible to determine confidently whether or not VEGF-A_165_b or VEGF-A_165_a cDNA is being amplified with isoform specific primers.

For standard quantitative PCR to be effective, isoform specific primers are necessary. Figure 2D shows the results of q-PCR on serial dilutions of VEGF-A_165_b and VEGF-A_165_a plasmids using both VEGF-A_165_b specific primers, forward ones spanning exons 5/7 and reverse primers spanning exons 7/8b, and VEGF-A_165_a specific primers (same forward, but reverse primers spanning exon 7/8a). These conditions were optimised for the q-PCR procedures to ensure specificity. Figure 2D shows that VEGF-A_165_a specific primers preferentially amplify VEGF-A_165_a but at very high amounts of VEGF-A_165_b, then amplification still occurred. Figure S1 shows the effect of mixing the templates. Addition of VEGF-A_165_a to VEGF-A_165_b or vice versa did not affect the amplification. Amplification curves and melt curves are shown in figure S2.

**Figure 3:**
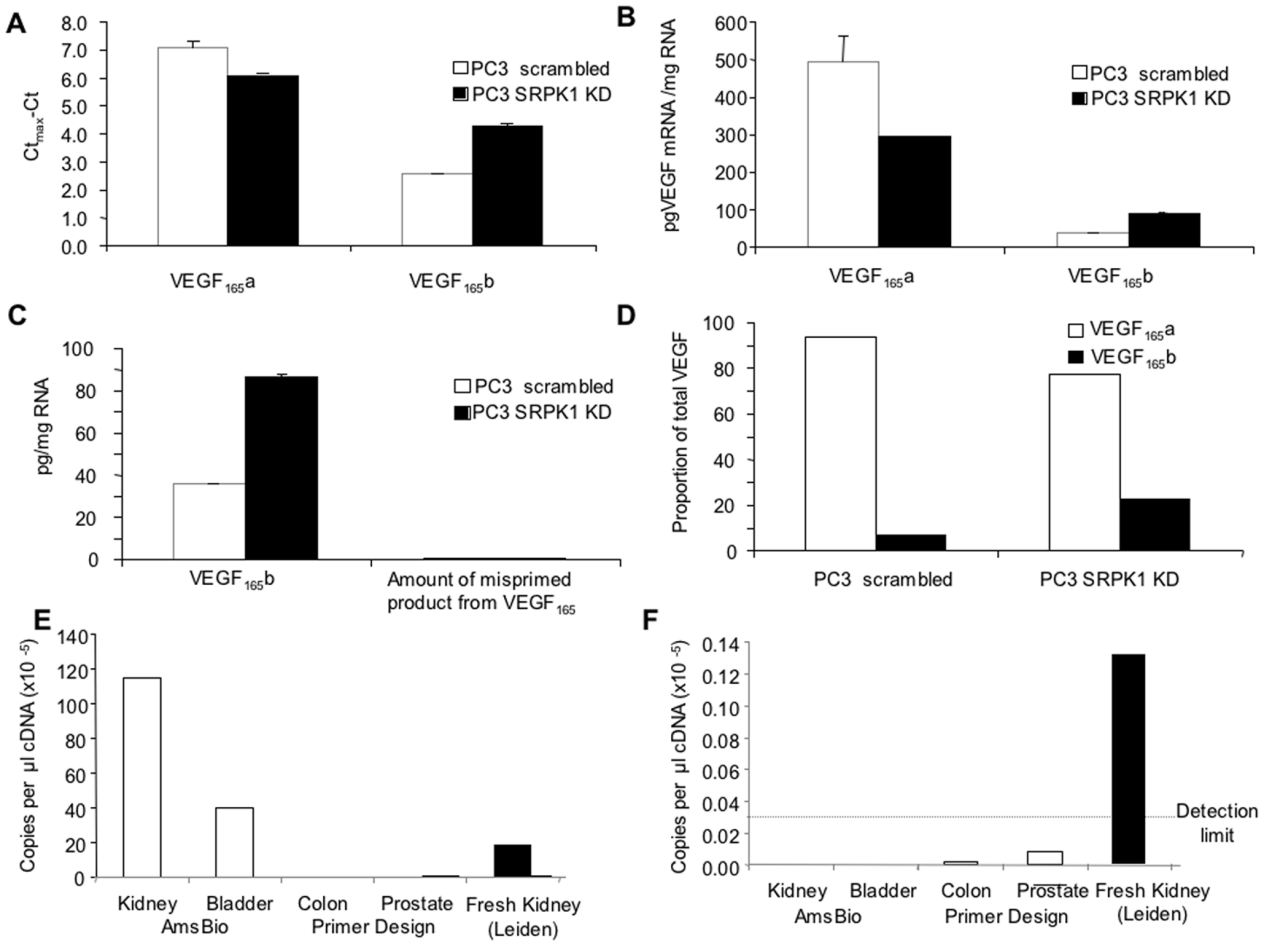
qRT-PCR using protocols shown in figure 2D and E can detect changes in splicing induced by splicing factor knockdown. A. C_t_max-C_t_ for cDNA extracted from prostate cancer (PC3) cells with lentiviral knockdown of SRPK1 or scrambled. B. Amount of VEGF calculated from standard curves in Figure 2. C. Amount of VEGF-A_165_b identified by Exon 8b primers (VEGF-A_165_b) or that calculated from mispriming of VEGF-A_165_a. D. Proportion of VEGF that is VEGF-A_165_a or VEGF-A_165_b in control and knockdown cells. Values are Mean±SEM (n = 2). 3E. qPCR for VEGF-A_165_a on commercially available cDNAs from 2 different companies (open bars) or cDNA reverse transcribed from freshly extracted human kidney RNA (solid bar). 3F qPCR for VEGF-A_165_b on commercially available cDNAs from 2 different companies (open bars) or cDNA reverse transcribed from freshly extracted human kidney RNA (solid bar).

The amplification of VEGF-A_165_b was 1481 fold less than the VEGF-A_165_a plasmid. The reverse experiment was also carried out showing that amplification of VEGF-A_165_a by VEGF-A_165_b specific primers was also possible at 1295 fold higher concentrations of VEGF-A_165_a than VEGF-A_165_b. Thus, as long as amplification of both products is carried out from templates containing the target sequences at known amounts it is possible to determine how much of the product, if any, could be due to mispriming. Figure 3A shows the cycle threshold (Ct) below maximum (35.8 for VEGF-A_165_a and 39.2 for VEGF-A_165_b) resulting from qPCR amplifications of cDNA generated from RNA extracted from PC3 cells transfected with control siRNA or siRNA to SRPK1 using the primers described in figure 2. There was a reduction in C_max_-C_t_ (i.e. increase in C_t_, and hence reduction in expression) for VEGF-A_165_a in the SRPK1 KD cells. In contrast VEGF-A_165_b expression was increased. Using the calibration curves from figure 2D and E, the amount of each isoform can be calculated. Figure 3B shows that there was an increase in VEGF-A_165_b from 35±0 to 86±4 pg per mg RNA, but a reduction in VEGF-A_165_a from 492±68 to 294±0pg per mg RNA (n = 2 each). As we know that the VEGF-A_165_b product can be amplified from VEGF-A_165_a template at an efficiency of 1295 fold lower using these primers, we can calculate that from 492pg/mg VEGF-A_165_a RNA a total of 0.38pg/mg VEGF-A_165_b could wrongly be assumed to be amplified. For the SRPK1 KD this would be 0.22pg/mg VEGF-A_165_b (figure 3C). This corresponds to 93 and 379 times less than actually detected respectively. Interestingly, these figures show that VEGF-A_165_b mRNA increased from 7±0.5% of total VEGF-A (in this case meaning exon 7 containing VEGF isoforms, calculated from the sum of the VEGF_165_a and VEGF_165_b amounts.) to 21.8±0.0% of total VEGF-A by knockdown of SRPK1.

We showed in figure 1 that the detection of VEGF_165_b isoforms can depend upon the quality of the cDNA. It was previously reported that PCR of cDNA purchased from commercial sources results in non-amplification of VEGF_165_b [23]. We repeated these experiments with a positive control – RNA extracted from fresh human kidneys from donors but not used for transplantation. Under these conditions, whereas GAPDH was amplified in all samples (not shown) VEGF-A_165_a was amplified to varying extents from commercial cDNAs and freshly prepared human kidney cDNA (figure 3E), VEGF-A_165_b was amplified from freshly prepared cDNA but not from the commercial cDNA (figure 3F) Interestingly, the relative amount of PCR product of VEGF-A_165_a varied between commercial sources.

### Detection of VEGF-A_165_b Protein by C Terminal Specific Antibodies

**Figure 4:**
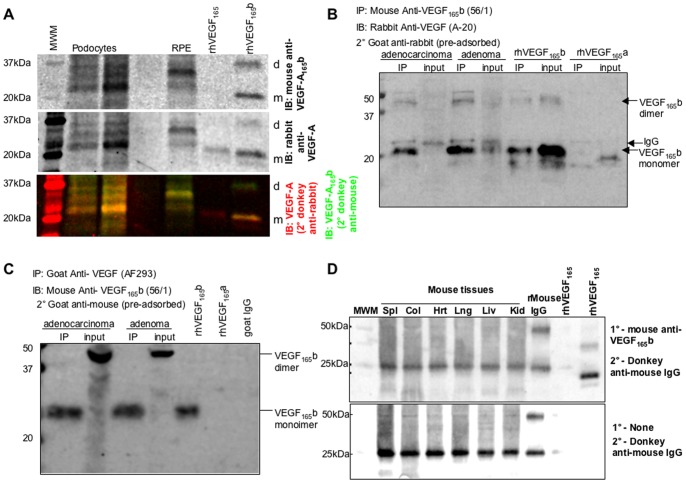
VEGF expression determined by Western blot and immunoprecipitation. A. Western blot using LiCor Odyssey to simultaneously image pan-VEGF and VEGF-A_165_b probed western blot. Two different podocyte samples, and a primary RPE sample were run on a gel and probed with antibodies to VEGF-A_165_b (mouse monoclonal anti-CTRSLTRKD, and 680nm-donkey anti-mouse, top image) and pan-VEGF (rabbit polyclonal anti-VEGF, and 800nm-donkey anti-rabbit, middle image). The bottom image is the pseudocoloured combined image (600nm green, 800nm red). Note the red VEGF_165_, but yellow VEGF-A_165_b. MWM = molecular weight marker. d = dimer, m = monomer. B. Protein extracted from human cell lines (adenoma and adenocarcinoma(AC)) subjected to immunoprecipitation (IP) for VEGF-A_165_b and immunoblotting (IB) for total VEGF-A. A clear strong band was seen in the IP for both cell types at ∼23kDa and ∼46kDa, consistent with the IP for recombinant human VEGF-A_165_b. A weaker band was seen in the input protein (not subjected to IP), and a second band slightly higher in the AC. A weak band at approximately 56kDa and 28kDa was seen in all lanes subjected to IP, including the VEGF-A_165_a band, but not seen in the recombinant human VEGF-A_165_b not subjected to IP, indicating that this is cross reactivity with the IgG. This band was clearly above the VEGF-A_165_b bands. C. Protein extracted from human cell lines (adenoma and adenocarcinoma(AC)) subjected to immunoprecipitation (IP) for VEGF-A and immunoblotting (IB) for VEGF-A_165_b. A clear strong band was seen in the IP for both cell types at ∼23kDa, the same size as recombinant human VEGF-A_165_b. In the input a band at ∼46Da was seen predominantly, for both cell types, labelled as VEGF-A_165_b dimers. D. Mouse tissues probed with VEGF-A_165_b antibody detect mouse IgG due to the secondary antibody. Top image, western blot of mouse tissues, recombinant mouse IgG or human VEGF-A_165_b or VEGF-A_165_b probed with mouse anti-CTRSLTRKD, and 680nm-donkey anti-mouse IgG. Bottom image blot of same tissues, probed without primary antibody. The same bands are seen in the mouse tissues. Spl = spleen, Col = colon, Hrt = heart, Lng = lung, Liv = liver, Kid = kidney.

In 2004, we developed a series of mouse monoclonal antibodies directed to the C-terminal nine amino acids of VEGF-A_165_b. One of these (MVRL56/1) is now commercially available from R&D systems (MAB3045) and AbCam (ab14994). MVRL56/1 has been used extensively to measure VEGF-A_165_b levels in human tissues [7], [9], [15], [29], [30]. Harris et al raised the possibility that this antibody may not be specific for VEGF. We used dual colour fluorescence imaging of western blots to show that this antibody detects VEGF isoforms (although not VEGF-A_165_a), shown in figure 4A. Human cell lysates from primary conditionally immortalised podocytes and from primary retinal pigmented epithelial cells, two cell types previously shown to express substantial VEGF-A_165_b levels [14], [31], were subjected to western blotting. The use of differentially labelled secondary antibodies was employed to coincidently detect VEGF isoforms using a pan-VEGF rabbit polyclonal antibody with a 680nm-fluorescent labelled donkey anti-rabbit secondary (shown as red), and the mouse anti-VEGF_xxx_b antibody with a 800nm-fluorescent labelled donkey anti-mouse secondary (shown as green). Recombinant human VEGF-A_165_a and VEGF-A_165_b were also run. The top image shows the 800nm monochrome fluorescence image, identifying three main bands at ∼25, 30 and 35kDa in both podocytes and RPE cells, no detection of VEGF-A_165_a, but clear detection of VEGF-A_165_b monomer and dimer. The middle image shows the 680nm image showing the anti-VEGF antibody, which shows exactly the same bands in the cells and rhVEGF-A_165_b, but also detects VEGF-A_165_a. The bottom image shows the merge of the two-colour fluorescent images to show that the bands detected by the VEGF-A_165_b antibody are absolutely coincident with the pan-VEGF antibody. The VEGF_165_b band may be slightly higher in the cells than the recombinant protein, which may be due to post-translational modification (i.e. different glycosylation), which may be cell type specific. The intensity of the bands between the two blots are similar, but, although we have previously shown that podocytes [31] and RPE cells [14] do express a substantial proportion of VEGF_165_b, we do not wish to imply that the Western blot is quantitative, as the antibodies are different, with different affinities, and therefore cannot be compared with each other in terms of relative levels of expression.

To further confirm that the anti-VEGF_165_b antibody detects human VEGF-A_165_b, protein was extracted from adenocarcinoma cells that show VEGF-A_165_b mRNA expression (figure 1D) and adenoma cells that we have previously shown have VEGF-A_165_b expression by RT-PCR, ELISA and western blotting, (AA/C1 colon adenoma cells). Five hundred micrograms of the protein was then subjected to immunoprecipitation using the mouse monoclonal antibody against VEGF-A_165_b (Clone 56/1). The immunoprecipitate (IP) and 50 µg of the crude extract were subjected to SDS-PAGE and immunoblotting using a rabbit polyclonal anti-pan-VEGF antibody (A20, Santa Cruz). Recombinant human VEGF-A_165_b and VEGF_165_a were used as positive and negative controls respectively. Figure 4B shows that the immunoprecipitation with an anti-VEGF-A_165_b antibody enriched the detection with the pan-VEGF antibody in both adenoma and adenocarcinoma cells, and specifically pulled down rhVEGF-A_165_b but not VEGF-A_165_a. Faint bands, higher than the VEGF monomer and dimer bands were seen in each of the IPs (including VEGF-A_165_b and VEGF-A_165_a recombinant protein IP) at 26kDa and 52kDa consistent with a weak affinity of the secondary goat anti-rabbit IgG for mouse IgG. This band was not seen in the protein not subjected to IP. To provide further proof, we carried out the reverse experiment, where total VEGF was immunoprecipitated from 500 µg of cell lysate, and then subjected to immunoblotting with the VEGF-A_165_b specific antibody (figure 4C). This showed that the IP with an anti-VEGF antibody enriched the detection with the anti-VEGF-A_165_b antibody of a VEGF monomer (at ∼23kDa), although interestingly not the dimer. We have previously noticed that AF293 tends to recognise the monomer of VEGF over the dimer. This experiment would appear to confirm this by precipitating preferentially the monomer. The anti-VEGF-A_165_b antibody detected VEGF-A_165_b but not VEGF-A_165_a or goat IgG (the immunoglobulin used to precipitate the VEGF).

While we [13], [14] and others [11] have published numerous western blots using this antibody on human tissue and others have used it on rat tissue [32] there are few examples of western blots published on mouse tissues or cell lines demonstrating VEGF-A_165_b expression. Recently, Harris et al used this VEGF-A_165_b antibody to try and determine VEGF-A_165_b expression in mouse tissues. They described protein detected in western blots with the mouse anti-VEGF-A_165_b antibody and an anti-mouse secondary antibody, including in protein from cells lines that should genetically lack VEGF-A_165_b. The bands were at 25kDa and 38kDa. This protocol has not previously been used, because the secondary antibody (an anti-mouse IgG) will pick up endogenous mouse IgG, which runs on a western blot at ∼25 and 48kDa, very similar to, but slightly higher than, recombinant VEGF-A_165_b. The experiment can be interpreted in three ways: either the secondary antibody (anti-mouse IgG) detects a protein in mouse cells and tissues directly, or that a protein in mouse cells and tissues is detected by mouse anti-human VEGF-A_165_b antibody that is then detected by the secondary antibody (anti-mouse IgG) of the same size as VEGF-A_165_b and mouse IgG, or thirdly, both the previous two events are occurring. The solution to this would have been to determine whether a secondary antibody used alone detected a band. Figure 4B shows a similar western blot to Harris et al, with mouse tissues demonstrating a clear band at ∼ 25kDa, similar to that found in the human tissue with the VEGF-A_165_b antibody. However, to underline the need for appropriate controls, we have also run mouse IgG as a negative control, and rhVEGF-A_165_b and rhVEGF-A_165_a as positive controls. It can be seen that the protocol detects mouse IgG. This could be either because the primary antibody detects mouse IgG or because the secondary, anti-mouse IgG detects mouse IgG. We therefore carried out the appropriate control western blot, which was to use no primary antibody. In this case the same band identified in the first blot was detected in all the mouse tissues, and the mouse IgG, but neither VEGF isoform, was detected. This simple control demonstrates that the mouse VEGF-A_165_b antibody should only be used on mouse tissue using protocols that prevent the secondary antibody from detecting endogenous protein and that a no-primary control should always be included when investigating mouse tissue.

## Discussion

These results demonstrate that investigation of VEGF-A_165_b expression, like all other robust experimental design, requires the use of appropriate positive and negative controls. The published evidence for human VEGF-A_165_b existence in normal human tissues is extensive and includes:

1. Cloning and sequencing of VEGF-A_165_b mRNA from normal human kidney [3], podocytes [13], retinal pigmented epithelial cells [14], and colon tissues [15].

2. Knockdown of VEGF-A_165_b by sequence specific siRNA reducing VEGF protein expression (detected by pan-VEGF ELISA) in cells in which VEGF-A_165_b is detected by PCR, but not in cells in which VEGF-A_165_b is a minor component [31].

3. Detection of VEGF-A_165_b by western blotting in human tissues by an antibody specific to the last nine amino acids of VEGF-A_165_b, which does not detect VEGF-A_165_a isoforms [7].

4. Detection of VEGF-A_165_b protein captured by pan-VEGF antibodies from cells expressing VEGF-A_165_b mRNA at greater levels than in those in which VEGF-A_165_b is a minor component [9], [15], [33].

5. Detection of VEGF in protein captured by VEGF-A_165_b antibodies from cells expressing VEGF-A_165_b mRNA at greater levels than in those in which VEGF-A_165_b is a minor component [6], [7].

6. Loss of VEGF-A_165_b expression in human kidneys from patients with mutations in the WT1 protein [8] and rescue of that expression by restoration of normal WT1 protein in cells isolated from those patients [13].

7. Increased expression of VEGF-A_165_b in conditions where lack of angiogenesis is associated with increased VEGF expression (systemic sclerosis) [10].

8. Pro-angiogenic effects of anti-VEGF-A_165_b antibodies in rodent developmental models [34].

There are still additional avenues that could be pursued to identify VEGF-A_165_b levels that do not rely on hybridisation of primers and amplification of cDNA, or detection by antibodies, including high throughput sequencing of unamplified cDNA from cells expressing VEGF-A_165_b, or mass spectrometry of all VEGF isoforms immunoprecipitated using generic anti-VEGF antibodies and these avenues are still being pursued. It is of course possible that the overwhelming published evidence on VEGF-A_165_b expression is all a series of unlikely concatenations of artefact. A protein detected by an antibody raised against the VEGF-A_165_b unique sequence, which does not detect VEGF-A_165_a would then be a spurious artefactual translation product of a mispriming event, a translation product which has profound and far reaching clinical potential in diseases, the pathophysiology of which is characterised either by VEGF dependent microvessel proliferation (e.g. malignancy, proliferative eye disease), or hyper-permeability (e.g. maculopathy, proteinuric renal disease). VEGF-A_165_b has been shown to be effective in *in vivo* models of these conditions. The artefactual protein would be both upregulated when VEGF is upregulated but angiogenesis is insufficient and downregulated in angiogenic conditions. The expression of this artefactual protein also predicts outcome of anti-VEGF therapy [35]. Alternatively, studies in which VEGF_xxx_b isoforms were not detected may have over-interpreted their results because of a failure to use appropriate controls [23]. Although it may be possible to clearly demonstrate an extraordinary concatenation of artefact, the experimental design to demonstrate such must include controls that clearly demonstrate this.

The examples given here – changing Mg^2+^ concentration, using different PCR machines or freeze thawing, are not intended to provide a specific protocol for VEGF-A_165_b amplification – in our experience, VEGF-A_165_b can be amplified in a PCR engine, or under lower Mg^2+^ concentrations, or on freeze thawed cDNA – but the conditions need to be optimised using positive controls, and even then a lack of product for any PCR reaction needs to be carefully interpreted in the light of possible experimental failures. For this reason multiple lines of evidence (e.g. ELISA, Western blot, PCR, qPCR) are ideally used for accurate identfiication VEGF-A_165_b levels (for instance see Varey et al [15]). For VEGF-A_165_b, known amounts of DNA encoding both VEGF-A isoforms are required to ensure that the PCR conditions are capable of detecting VEGF-A_165_a and VEGF-A_165_b, as demonstrated in every publication on VEGF-A_165_b from our laboratory since 2002. To understand and recognise the detection limits of the experimental procedure is key. Failure to use these positive controls means the negative findings on VEGF-A_165_b expression are not interpretable. Indeed we were very mindful of the need for appropriate controls when VEGF-A_165_b was first identified using positive controls to ensure that mispriming did not occur [3].

A previous study investigating conservation of alternative splicing of VEGF across species found that VEGF-A_165_b was more highly expressed (examined by qPCR) in human than cat, than rabbit, than rat, and that mice had the lowest level of expression of all mammalian species investigated [22]. The use of mouse tissues and cells to investigate human VEGF_xxx_b isoforms is understandable due to the availability of transgenic models, and the identification of mouse VEGF-A_165_b and development of antibodies that specifically detect these isoforms will be valuable additions to the field, and are in process. It will be particularly interesting to examine VEGF-A_165_b expression in mouse VEGF knockout tissues. Harris et al tried to investigate VEGF-A_165_b expression in mouse VEGF knockout fibrosarcoma cell lines. However, the use of cancer cells (low VEGF_xxx_b expression relative to normal cells) from mice (lowest VEGF_xxx_b expression of all eutherian species investigated) makes it unlikely that they would have detected VEGF_xxx_b isoforms. The lack of positive control experiments to show that their PCR conditions were capable of detecting VEGF_xxx_b isoforms makes it even more unlikely that they could extrapolate their results to normal mouse or human tissues. Finally the use of a secondary antibody against mouse immunoglobulin to detect the mouse anti-VEGF-A_165_b immunoglobulin guarantees a false positive response in the VEGF knockout mice. Therefore, while unequivocal demonstration of mouse VEGF_xxx_b protein and mRNA expression is highly anticipated, current published evidence against its existence is much weaker than that in favour of such existence.

The evidence for VEGF_xxx_b expression in human tissue on the other hand is overwhelming. The mechanism of action, regulation of splicing, role in disease, and evolutionary derivation are all interesting areas of research. For instance, we would draw attention to the elegant experiments published by Ballmer Hofer et al demonstrating differential signalling dependent on neuropilin-1 binding [36]. While we do not disagree with the statement that further examination of aspects of VEGF-A_165_b biology is required, we would suggest that questioning the physiological existence of VEGF-A_165_b isoforms based on experimental design that dos not provide controls for the felicity of experimental conditions, and that a conclusion that anti-angiogenic isoforms are not highly expressed in human or mouse tissues, is not supported by currently published results.

### Conclusions

Detection of alternative splice variants of VEGF needs to be carried out with experimental design that includes appropriate use of positive and negative controls. These include sequencing PCR products where possible, including positive control DNA - either plasmids, or cDNA from cells known to express VEGF-A_165_b - human primary RPE cells, human conditionally immortalised podocytes, or human adenoma (AA/C1) or adenocarcinoma (10C) cells. The interpretation of negative results in the absence of positive controls is not possible and conclusions drawn from such experiments need to be challenged. The existence of VEGF_xxx_b isoforms can be, and has been, clearly demonstrated from human tissues, and there are multiple lines of evidence supporting its existence, function and physiological role in rodents and other eutherian species. More research is clearly required to understand the role of VEGF-A_165_b in human disease and in animal models of disease.

## Supporting Information

Figure S1 A. qPCR using VEGF-A_165_a specific primers on a mixture of VEGF-A_165_a and VEGF-A_165_b plasmid at varying mixtures .B. The effect of adding in VEGF_165_b to VEGF_165_a template did not affect the sensitivity (slope) of the qPCR reaction. C. qPCR using VEGF-A_165_b specific primers on a mixture of VEGF-A_165_a and VEGF-A_165_b plasmid at increasing doses. D. Again, the effect of adding in VEGF_165_a to VEGF_165_b template did not affect the sensitivity (slope) of the qPCR reaction.
(DOCX)

Figure S2 **Q-PCR for VEGF_165_b and VEGF_165_b using isoform specific primers.** A. Fluorescence intensity curves for qPCR for VEGF_165_a using isoform specific primers. B. Fluorescence intensity curves for qPCR for VEGF_165_b using isoform specific primers. C. Melt curve for VEGF_165_a. D. Melt curve for VEGF_165_b
(DOCX)
