# Supplementary material for: Detection of VEGF-Axxxb Isoforms in Human Tissues
Source: PLoS One. 2013 Jul 31;8(7):e68399. doi: 10.1371/journal.pone.0068399 (PMC3729684; doi:10.1371/journal.pone.0068399)
Supplement: Figure S1 — A. qPCR using VEGF-A165a specific primers on a mixture of VEGF-A165a and VEGF-A165b plasmid at varying mixtures .B. The effect of adding in VEGF165b to VEGF165a template did not affect the sensitivity (slope) of the qPCR reaction. C. qPCR using VEGF-A165b specific primers on a mixture of VEGF-A165a and VEGF-A165b plasmid at increasing doses. D. Again, the effect of adding in VEGF165a to VEGF165b template did not affect the sensitivity (slope) of the qPCR reaction. (DOCX) [file pone.0068399.s001.docx]

Figure S1. A. qPCR using VEGF-A_165_a specific primers on a mixture of VEGF-A_165_a and VEGF-A_165_b plasmid at varying mixtures .B. The effect of adding in VEGF_165_b to VEGF_165_a template did not affect the sensitivity (slope) of the qPCR reaction. C. qPCR using VEGF-A_165_b specific primers on a mixture of VEGF-A_165_a and VEGF-A_165_b plasmid at increasing doses. D. Again, the effect of adding in VEGF_165_a to VEGF_165_b template did not affect the sensitivity (slope) of the qPCR reaction.
