# Supplementary material for: Detection of VEGF-Axxxb Isoforms in Human Tissues
Source: PLoS One. 2013 Jul 31;8(7):e68399. doi: 10.1371/journal.pone.0068399 (PMC3729684; doi:10.1371/journal.pone.0068399)
Supplement: Figure S2 — Q-PCR for VEGF165b and VEGF165b using isoform specific primers. A. Fluorescence intensity curves for qPCR for VEGF165a using isoform specific primers. B. Fluorescence intensity curves for qPCR for VEGF165b using isoform specific primers. C. Melt curve for VEGF165a. D. Melt curve for VEGF165b (DOCX) [file pone.0068399.s002.docx]

Figure S2. Q-PCR for VEGF_165_b and VEGF_165_b using isoform specific primers. A. Fluorescence intensity curves for qPCR for VEGF_165_a using isoform specific primers. B. Fluorescence intensity curves for qPCR for VEGF_165_b using isoform specific primers. C. Melt curve for VEGF_165_a. D. Melt curve for VEGF_165_b.
